# Supplementary material for: Suppressed electronic contribution in thermal conductivity of Ge2Sb2Se4Te
Source: Nat Commun. 2021 Dec 10;12:7187. doi: 10.1038/s41467-021-27121-x (PMC8664948; doi:10.1038/s41467-021-27121-x)
Supplement: Supplementary file 1 — Supplementary Information [file 41467_2021_27121_MOESM1_ESM.pdf]

Suppressed electronic contribution in thermal conductivity of $Ge_2Sb_2Se_4Te$

Kiumars Aryana[1], Yifei Zhang[2], John A. Tomko[1], Eric R. Hoglund[3], David H. Olson[1], Joyeeta Nag[4], John C. Read[4], Carlos Ríos[5,6], Juejun Hu[2], and Patrick E. Hopkins[1,3,7,*]

[1]Department of Mechanical and Aerospace Engineering, University of Virginia, Charlottesville, VA 22904, USA

[2]Department of Materials Science and Engineering, Massachusetts Institute of Technology, Cambridge, MA, USA

[3]Department of Materials Science and Engineering, University of Virginia, Charlottesville, VA 22904, USA

[4]Western Digital Corporation, San Jose, CA 95119, USA

[5]Department of Materials Science and Engineering, University of Maryland, College Park, MD 20742, USA

[6]Institute for Research in Electronics and Applied Physics, University of Maryland, College Park, MD 20742, USA

[7]Department of Physics, University of Virginia, Charlottesville, VA 22904, USA

[*]phopkins@virginia.edu

Supplementary Note 1

STEM-EDS was performed using a 400 pA beam current and the results are shown in Figure S1. The annular darkfield image in Figure S1(a) shows multiple equiaxed and non-equiaxed grains. The composition of the various grains do not have differing compositions in anyway correlated to the morphologies in the darkfield signal, as shown in Figure S1(b-e). A compositional gradient is present across the thickness of the films, as shown in Figure S1(h). The gradient may in part be from x-ray absorption affects that are thickness dependent or could be a result of growth conditions. It is striking that such a large gradient is present, and the gradient may explain the preferential non-equiaxed grain morphology being present at the bottom of the film and not the top.

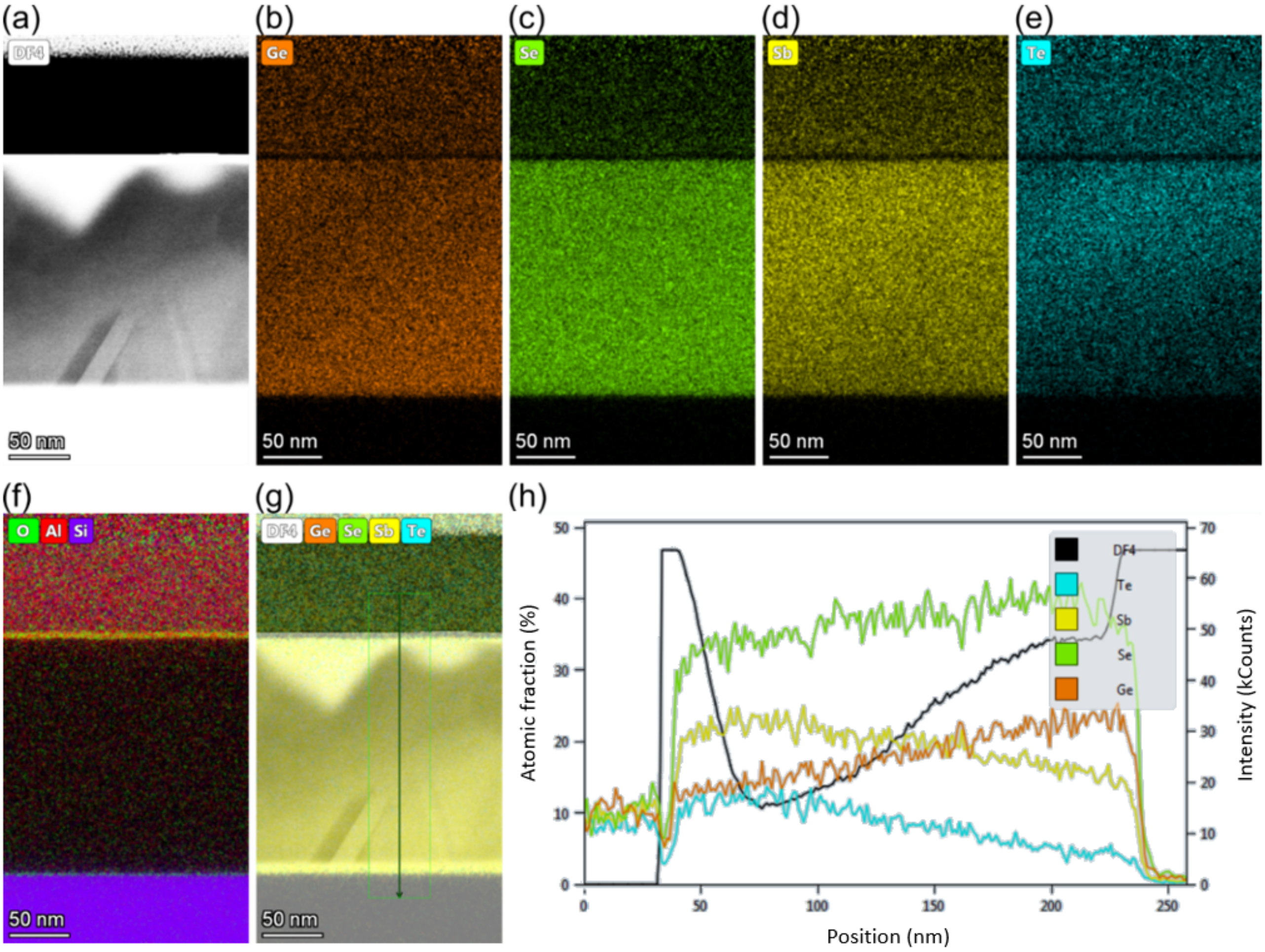

Supplementary Figure 1. STEM-EDS spectrum image with (a) diffraction contrast from an annular darkfield detector showing multiple grains with uniform (b) Ge, (c) Se, (d) Sb, and (e) Te compositions. Observation of signatures of other elements in the Al transducer is associated with the noise in the system and artifact of measurement. The Si and Al layers are shown in (f) along with thin oxides present at each interface. The composite image of (a-e) is shown in (g), where a line profile is indicated. The quantitative compositions along the line profile are shown in (h).

Supplementary Note 2

In order to avoid any possible diffusion or reaction between GSST and Pt, we deposited a thin layer of Al_2O_3 between the two layers. In this section, we demonstrate that the addition of this layer has minimal impact on our measured thermal conductivity. For this, although Al_2O_3 is very thin, since it has a heat capacity close to that of Pt, the effect of its heat capacity may interfere with our result. In order to account for this, we use a 4-layer model and treat Al_2O_3 as a separate layer instead of an interface. However, according to our result, if the heat capacity of Al_2O_3 changes by a factor of two, it has minimal impact on the measured thermal conductivity. Although variations in the heat capacity result in the change in the Y-axis intercept (interfacial thermal resistance), it does not change the slope of the fitted line. This is because due to the large interfacial resistance between Al_2O_3 and GSST, the effect of heat capacity is negligible. This agrees with our thermal conductivity measurements as a function of temperature, where we observe no change in the thermal conductivity of 20 nm GSST film which stems from the fact that the interfacial thermal resistance between Al_2O_3 and the GSST is the dominant resistor rather than the GSST film it self.

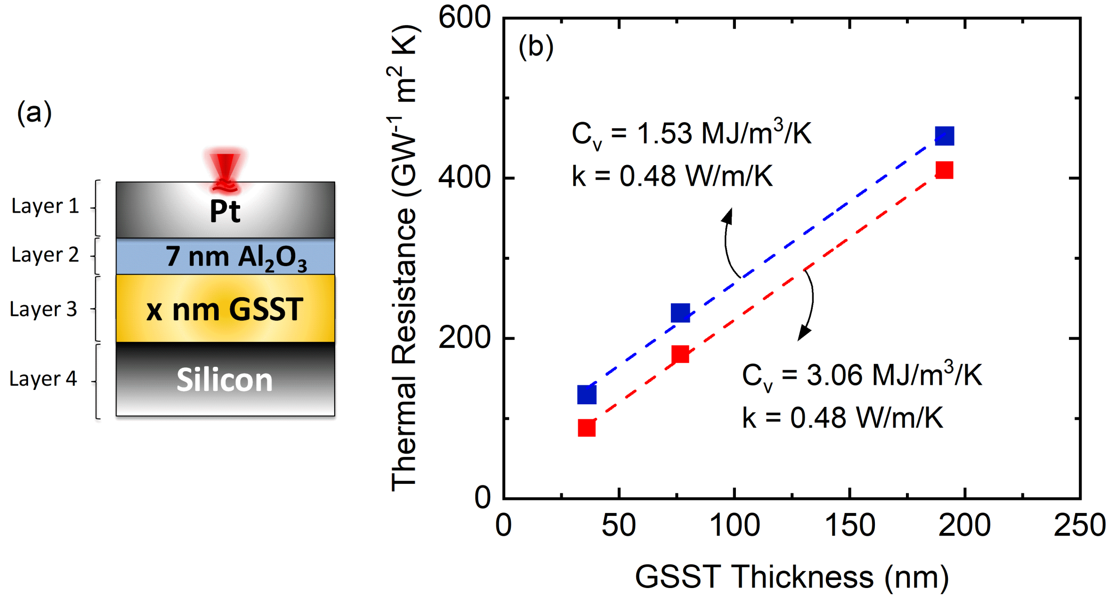

Supplementary Figure 2. (a) The schematic showing the configuration of the layers in our thermal conductivity measurements. (b) Thermal resistance as a function of GSST thickness assuming two different specific heat for the Al_2O_3.
